# C5 Nerve Palsy After Posterior Instrumentation and Decompression in Cervical Spine Surgery: A Review of the Literature

**DOI:** 10.7759/cureus.84430

**Published:** 2025-05-19

**Authors:** Konstantinos Zygogiannis, Pavlos Gerasimidis, Spyridon Komaitis, Savvas Moschos, Georgios C Thivaios, Aikaterini Tsatsaragkou, Dimitrios Koulalis

**Affiliations:** 1 Orthopaedics - Scoliosis and Spine, KAT General Hospital, Athens, GRC; 2 Orthopaedics and Traumatology, Attikon Hospital, Athens, GRC; 3 Neurological Surgery, Centre for Spinal Studies and Surgery, Queens Medical Centre, Nottingham University Hospitals, Nottingham, GBR; 4 Trauma and Orthopaedics, Laiko General Hospital of Athens, Athens, GRC; 5 Family Medicine, General Practice, and Flying Doctor, National Emergency Aid Centre, Athens, GRC; 6 Orthopaedics and Traumatology, Attikon University Hospital, Athens, GRC

**Keywords:** c5 nerve root palsy, cervical spine surgery, posterior cervical decompression, posterior spine fixation, spine surgery complications

## Abstract

C5 nerve palsy is a well-documented postoperative complication of cervical spine surgery, particularly following posterior decompression and fixation procedures. With incidence rates reported between 4% and 30%, it poses significant clinical challenges due to its impact on upper limb function and patient quality of life. Typically emerging within one to four weeks post-surgery, C5 palsy is marked by deltoid and biceps muscle weakness and sensory deficits in the C5 distribution. The pathogenesis is multifactorial, involving spinal cord shift, reperfusion injury, and foraminal stenosis, with surgical factors such as decompression extent and alignment correction also contributing. While most cases respond favorably to conservative management, including physical rehabilitation, some patients experience prolonged recovery or residual deficits. Recovery rates range from 71% to 96%, emphasizing the importance of individualized rehabilitation protocols. Anatomical predispositions, preoperative conditions, and surgical techniques are critical in both the development and management of this complication. Continued research is needed to refine predictive models and tailor treatment strategies based on patient-specific and procedural variables. This review study aims to collect available data and summarize the information available in the medical community according to the Preferred Reporting Items for Systematic Reviews and Meta-Analyses (PRISMA) guidelines.

## Introduction and background

C5 nerve palsy is a recognized postoperative complication subsequent to cervical spine surgery, particularly after procedures involving posterior decompression and fixation strategies. With reported incidences ranging from 4% to 30%, the condition presents significant clinical challenges due to its impact on upper limb function and quality of life for patients [1-3]. Characterized by weakness in the deltoid and biceps muscles, as well as sensory disturbances in the C5 nerve root distribution, the onset of symptoms typically occurs within one to four weeks postoperatively, creating a pressing need for timely and appropriate management strategies [4-6].

The pathogenesis of C5 nerve palsy is multifaceted, encompassing a variety of mechanisms including segmental spinal cord shift, reperfusion injury, and foraminal stenosis, alongside considerations regarding surgical factors such as the extent of decompression and cervical alignment correction [7-9]. As such, a comprehensive understanding of both anatomical and physiological elements is critical in mitigating risks associated with C5 palsy.

Management approaches are often stratified into conservative and surgical methods, with most patients benefiting from conservative rehabilitation strategies that focus on physical therapy to enhance recovery and regain muscle strength [3,10,11]. Despite the generally favorable prognosis, where recovery rates are reported between 71% and 96%, some individuals may experience protracted recovery timelines or persistent deficits necessitating further intervention [12, 13]. Consequently, effective rehabilitation protocols, encompassing a combination of strength training and supportive therapy, are imperative to optimize outcomes for patients afflicted by this complication [7, 14, 15].

## Review

Materials and methods

**Figure 1:**
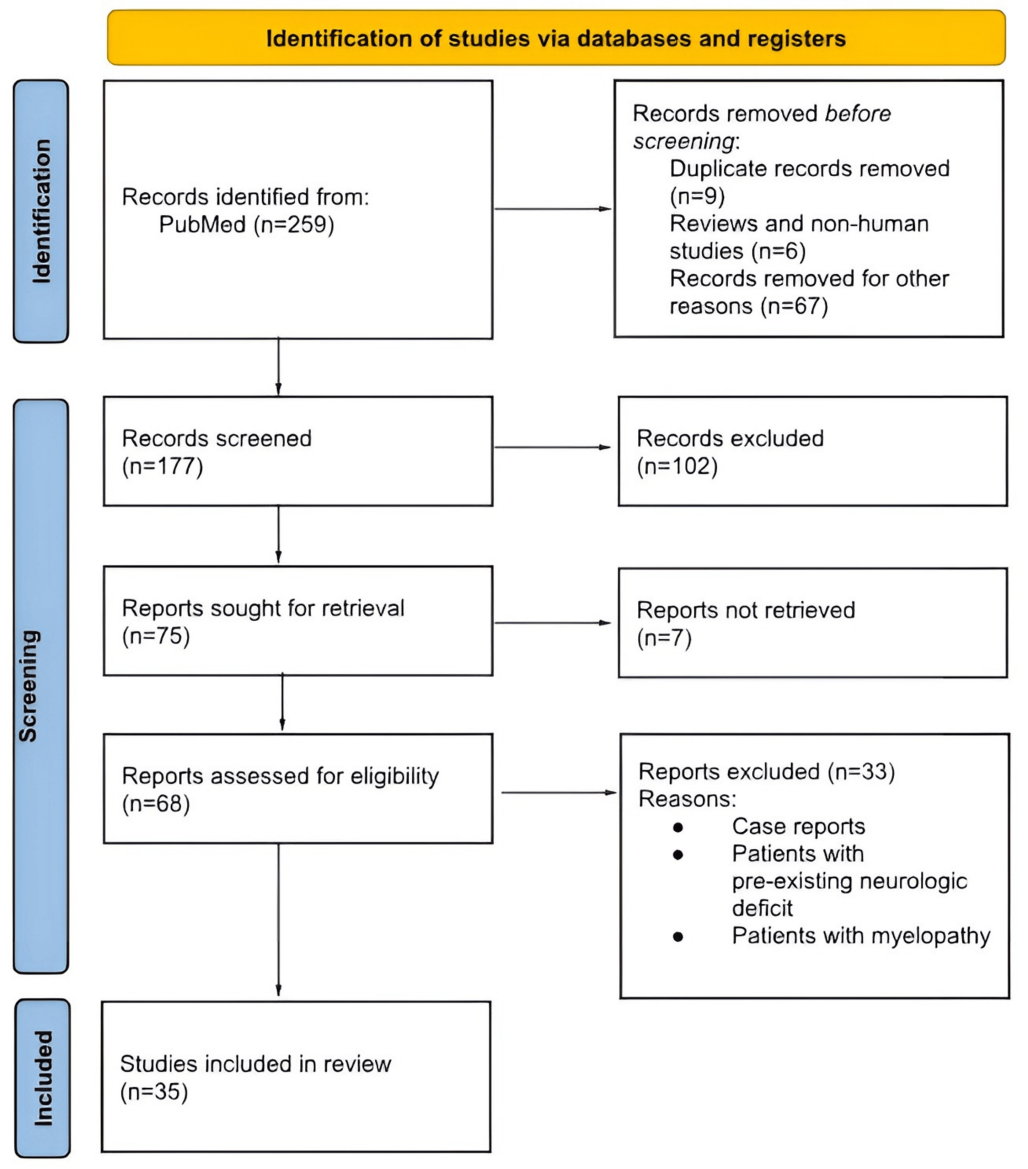
Flowchart of the review

This systematic review was conducted in accordance with the Preferred Reporting Items for Systematic Reviews and Meta-Analyses (PRISMA) guidelines (Figure 1). A comprehensive literature search was performed across databases including PubMed, Embase, and Cochrane Library to identify studies reporting on the incidence, pathogenesis, risk factors, and management of C5 nerve palsy following cervical spine surgery. Keywords such as spine surgery complications, cervical spine surgery, posterior cervical decompression, C5 nerve root palsy, and posterior spine fixation were used in various combinations. Studies published solely in English were considered, and both prospective and retrospective clinical studies, as well as relevant review articles, were included. Titles and abstracts were screened independently by two reviewers, with full-text articles assessed for eligibility based on predefined inclusion and exclusion criteria.

Incidence of C5 palsy

Reported rates of C5 palsy in existing literature vary considerably, underscoring the complexity and variability of clinical outcomes in cervical spine surgeries. Generally, the incidence has been documented to range from approximately 4% to as high as 30%, depending on surgical techniques and patient demographics [3,13]. For instance, a comprehensive meta-analysis that included data from over 13,000 patients highlighted that the overall prevalence of C5 palsy following cervical surgery is particularly notable among those undergoing laminoplasty and laminectomy procedures [3]. In another study, Bydon et al. reported an incidence of 14.5% after posterior foraminotomy [12]. Additionally, Oh et al. observed a stark difference in incidence rates based on surgical approach, noting a higher occurrence in posterior surgeries compared to anterior interventions [13]. The variability in reported rates can also be attributed to the types of surgeries performed. For instance, patients with ossification of the posterior longitudinal ligament (OPLL) demonstrated a higher incidence of C5 palsy of up to 14% during laminoplasty [14]. Specific surgical methods, such as open-door laminoplasty or laminectomy, exhibit differing rates of C5 palsy, emphasizing the need for careful consideration of surgical approach in risk management.

Risk factors and demographic trends

The factors influencing the development of C5 palsy are multifaceted. Several studies have identified demographic and clinical variables as significant risk factors. For instance, male patients have been consistently identified as being at a higher risk compared to female patients, with incidence rates reported at 22% for males compared to 5.2% for females [13]. Other demographic factors, including age and baseline cervical spine conditions such as OPLL and cervical foraminal stenosis, have also been identified as contributing elements [15]. Various surgical parameters further impact the likelihood of developing C5 palsy. The width of the C4-C5 intervertebral foramen appears to be a critical factor, with a narrower foraminal space correlated with a higher incidence of nerve root palsy [7]. Surgical techniques involving excessive retraction during decompression, particularly those that alter spinal alignment or add asymmetry to decompression effort, significantly elevate risk [7,10,12]. Additionally, intraoperative factors, such as the angle of the vertebral arch and the methodology employed in laminoplasty, have been linked to a greater risk due to potential uneven strain on cervical structures [10,16]. Furthermore, studies suggest that radiological assessments, such as the measurement of preoperative spinal cord rotation, may serve as predictive factors for postoperative nerve complications [7]. This indicates that careful preoperative evaluation and planning could play a crucial role in mitigating risks for susceptible populations (Table 1).

**Table 1:** Summary of C5 palsy incidence, risk factors, and demographic trends OPLL: ossification of the posterior longitudinal ligament

Category	Factor	Details	Key References
Incidence	Overall incidence	4% to 30%, depending on surgical techniques and patient factors	Wang et al. (2017) [3], Oh et al. (2019) [13]
	Laminoplasty and laminectomy	Higher prevalence of C5 palsy	Wang et al. (2017) [3]
	Posterior foraminotomy	14.5% incidence reported	Bydon et al. (2014) [12]
	Posterior vs. Anterior surgeries	Higher incidence in posterior approaches	Oh et al. (2019) [13]
	OPLL patients undergoing laminoplasty	Incidence up to 14%	Gu et al. (2014) [14]
Risk Factors	Sex	Higher risk in males (22%) compared to females (5.2%)	Oh et al. (2019) [13]
	Age and baseline cervical pathology	Older age, OPLL, and cervical foraminal stenosis increase risk	Lee et al. (2017) [15]
	Narrow C4-C5 intervertebral foramen	Associated with increased risk of nerve root palsy	Chugh et al. (2015) [7]
	Excessive surgical retraction and asymmetry during decompression	Increases mechanical stress and risk	Bydon et al. (2014) [12], Baba et al. (2016) [10], Chugh et al. (2015) [7]
	Surgical technique (angle, method in laminoplasty)	Certain techniques may cause uneven strain and elevate risk	Baba et al. (2016) [10], Frazzetta et al. (2024) [16]
	Preoperative spinal cord rotation	Predictive factor for postoperative C5 palsy	Chugh et al. (2015) [7]

Anatomy and pathophysiology

Relevant Cervical Spine Anatomy (C4-C6)

The C4-C6 region of the cervical spine is critical due to its anatomical configuration, which encompasses essential structures such as the cervical vertebrae, intervertebral discs, and nerve roots, particularly the C5 nerve root. The intervertebral foramina at C4-C5 and C5-C6 are pivotal as they house the exiting spinal nerves. A significant aspect of the anatomy is that C5 is particularly sensitive to injury due to its anatomical positioning and the surrounding soft tissues [1-3]. It is also subjected to mechanical stress, especially given that the cervical lordotic apex is located at C5, leading to significant motion-related forces at this level [2,4]. Additionally, foraminal stenosis at these levels can contribute to nerve root compression, which may exacerbate conditions leading to postoperative complications such as C5 palsy [5,15].

Pathophysiological Mechanisms Behind C5 Palsy

The development of C5 nerve palsy following cervical decompression surgery has been attributed to several pathophysiological mechanisms. One prominent theory suggests that traction of the C5 nerve root occurs due to the posterior displacement of the spinal cord following decompression procedures [3,5]. This phenomenon, known as the "tethering effect," occurs when the spinal cord shifts posteriorly, which can overstretch or impinge upon the C5 nerve root within the neuroforamen [5,9]. Studies indicate that pre-existing conditions, like foraminal stenosis or subclinical nerve root compression, further predispose patients to develop C5 palsy postoperatively [2,6].

Deficiencies in blood supply to the nerve root during surgical manipulation have also been hypothesized as a contributing factor [7,8]. Moreover, the timing of neurological injury relative to the decompression intervention is critical. The C5 nerve appears more vulnerable compared to other cervical nerves, potentially linked to its unique neuroanatomical relationships with adjacent vascular structures [1,10]. As a result, this idiosyncrasy necessitates that surgeons exercise heightened awareness during procedures involving this nerve root to mitigate risks [2,11].

Furthermore, additional risk factors such as specific surgical approaches, including double-door laminoplasty versus other decompression techniques, have been analyzed. Certain surgical angles and techniques can increase the mechanical stress on the C5 nerve root, compounding the risk of palsy [9,10]. Operative strategies that emphasize protective measures for the C5 nerve root during decompression are also being evaluated as a means to reduce the incidence of this complication (Table 2) [5,7].

**Table 2:** Anatomical, pathophysiological, and surgical considerations regarding C5 nerve palsy

Category	Factor	Details	Key References
Anatomy	C5 nerve root course	Short distance from dural origin to foramen → limited mobility	Imagama et al. (2010) [8], Guzman et al. (2014) [5]
	Intervertebral foramen at C4-C5	Narrow foramen + rigid foraminal ligaments → increased risk of tethering	Nassr et al. (2012) [2], Wang et al. (2017) [3]
	Cervical lordotic apex	Typically at C4–C5 → increased mechanical stress at this level	Ezra et al. (2018) [4] Nassr et al. (2012) [2]
Pathophysiology	Tethering effect	Posterior cord shift → traction on fixed C5 nerve root	Wang et al. (2017) [3], Krätzig et al. (2017) [9]
	Ischemia-reperfusion injury	Microvascular injury from decompression/reperfusion	Imagama et al. (2010) [8] Baba et al. (2016) [10]
	Segmental spinal cord disorder	Anterior horn cell damage at C5 segment → motor deficit	Baba et al. (2016) [10]
	Pre-existing foraminal stenosis	Increases vulnerability to traction injury	Li et al. (2022) [6], Guzman et al. (2014) [5]
Surgical Considerations	Technique (e.g., laminoplasty vs. laminectomy)	Posterior decompression affects degree of spinal cord shift	Baba et al. (2016) [10] Kim et al. (2014) [11]
	Lack of prophylactic foraminotomy	Associated with increased risk of nerve root compression	Chugh et al. (2015) [7]
	Intraoperative neuromonitoring	Suggested as a preventive strategy	Guzman et al. (2014) [5]

Proposed mechanisms of pathogenesis

Segmental Spinal Cord Shift

One widely accepted theory suggests that the posterior shift of the spinal cord during decompression operations places significant tension on the C5 nerve root, leading to injury. In this context, the C5 nerve root can become tethered against adjacent anatomical structures due to altered biomechanics following surgery [3,14]. The anisotropy of the nerve root makes it particularly susceptible to traction injuries that can arise from sudden shifts or movements of the spinal cord during surgical manipulation [17,18].

Reperfusion Injury

Reperfusion injury is cited as a potential mechanism for C5 palsy, particularly in scenarios involving ischemia during surgical procedures. When blood flow is restored rapidly after a period of reduced perfusion, it can lead to cellular damage due to oxidative stress. This phenomenon has been observed in various surgical contexts, where localized ischemia followed by reperfusion can adversely affect nerve integrity, particularly those that are already compromised by pre-existing stenotic conditions [14,19]. In the case of C5 palsy, this ischemia-reperfusion dynamic could lead to further injury to the C5 nerve root and associated motor pathways [14,20].

Foraminal Stenosis

Foraminal stenosis at the C4-C5 and C5-C6 levels is another recognized contributor to C5 nerve palsy. Studies suggest that pre-existing foraminal stenosis may predispose patients to postoperative complications because the C5 nerve root can become severely compromised during the decompression process [10,21]. As the foraminal space is narrowed, any surgical manipulation could further compromise the nerve root's blood supply and mechanical stability, thereby increasing the likelihood of postoperative palsy [15,18].

Surgical factors

Extent of Decompression

The extent and nature of decompression play critical roles in the incidence of C5 palsy. Techniques that involve aggressive decompression, particularly of multiple levels, may inadvertently exacerbate the mechanical stress on the C5 nerve root [12,22]. There is an ongoing debate regarding the balance between adequate decompression and minimizing the risk of nerve root injury, illustrating the need for careful surgical planning and execution [3,19]. Cases demonstrate that more extensive lateral decompressions or inadequate bony decompression may lead to higher incidences of palsy, necessitating a tailored approach for each patient [15,23].

Alignment Correction

Surgical correction of cervical alignment has also been linked with pathogenesis. For instance, attempts to achieve ideal lordotic alignment may inadvertently place additional strain on the C5 nerve root due to changes in the biomechanics of the cervical spine [16,24]. The C5 nerve root, located at the apex of the cervical lordosis, may be particularly susceptible to how alignment corrective measures are implemented. Understanding the implications of spinal alignment during decompression strategies could provide insights into reducing incidence rates of potential neurologic deficits [3,11].

Clinical presentation and timing of C5 palsy

The typical presentation of C5 nerve palsy involves weakness in the deltoid and biceps brachii muscles, manifesting as difficulty with shoulder abduction and elbow flexion. Patients often report sensory disturbances in the regions innervated by the C5 nerve, including areas of the upper arm and shoulder [14,19,25]. The onset of symptoms usually occurs within a week following surgery; however, a minority of cases may present 2 to 4 weeks postoperatively, reflecting potential delayed neurological recovery or injury [14,15].

Differentiation from other neurological complications

Differentiating C5 palsy from other neurological issues is crucial, as patients may experience coinciding conditions such as brachial plexus injuries, which can also present similar motor deficits. Relevant distinctions include the location and nature of weakness; C5 palsy typically spares hand functions, which are innervated by more distal nerve roots, while brachial plexus injuries generally encompass broader deficits across multiple nerve roots [19]. Furthermore, C5 palsy often has a hemilateral presentation, whereas brachial plexus injuries may show more diffuse involvement [19]. To accurately differentiate between these conditions, clinicians must conduct a thorough neurological examination focused on muscle strength testing and sensory assessment specific to the C5 nerve distribution. Pain patterns can also provide insight; while C5 palsy may present with minimal pain, brachial plexus injuries often involve significant discomfort.

Diagnostic workup

The diagnostic workup for C5 palsy after cervical spine surgery involves a combination of thorough clinical evaluation, advanced imaging modalities, and neurophysiological testing. A comprehensive clinical assessment is foundational, with emphasis on detailed history-taking and focused neurological examination to evaluate muscle strength, particularly in the deltoid and biceps, as well as sensory changes in the C5 dermatome [8,14]. Imaging studies such as MRI and CT scans are critical in detecting structural abnormalities like foraminal stenosis, disc herniation, or ossification of the posterior longitudinal ligament (OPLL) that could predispose patients to C5 nerve root compression [12,14,25]. MRI can also help rule out spinal cord signal changes, while CT is particularly useful in evaluating bony structures. Neurophysiological studies, including electromyography (EMG) and nerve conduction studies (NCS), provide objective evidence of C5 nerve dysfunction by assessing the electrical activity in C5-innervated muscles and differentiating between peripheral neuropathies, radiculopathies, or central lesions [3,18]. These studies not only confirm the diagnosis but also help quantify the severity of nerve damage and can be instrumental in guiding prognosis and management.

Conservative vs. surgical management

C5 nerve palsy is frequently managed conservatively, given the often self-limiting nature of the condition and high spontaneous recovery rates. Several studies have demonstrated favorable outcomes with conservative measures, reporting recovery in approximately 71% to 96% of cases [5,26]. Conservative management typically involves physical therapy aimed at strengthening the deltoid and biceps, improving range of motion, and preventing secondary complications such as joint contractures [27,28]. Analgesics, corticosteroids, and anti-inflammatory medications are often prescribed to control pain and inflammation. A study by Acharya and Palukuri reported notable improvements in motor recovery following a structured physiotherapy program, emphasizing the potential for non-operative rehabilitation even in cases of moderate muscle weakness. As such, conservative treatment is generally considered the first-line approach, especially in patients without progressive neurological deficits or structural abnormalities. Surgical intervention may be indicated when symptoms are severe, persist despite conservative efforts, or if imaging reveals persistent foraminal stenosis or unaddressed nerve root compression [17]. However, surgical re-intervention remains controversial, as evidence suggests that while decompression may resolve structural issues, it does not consistently lead to improved functional outcomes and may carry a risk of further nerve injury (Table 3) [14].

**Table 3:** Conservative vs. surgical management of C5 palsy

Management	Description	Outcomes	Key References
Conservative	Physical therapy, analgesics, observation	71-96% recovery; muscle strength improves over months	Saoud et al. (2013) [26], Guzman et al. (2014) [5], Acharya et al. (2016) [17]
	Rehabilitation targeting deltoid/biceps strength	Prevents complications; enhances motor recovery	Kubota et al. (2021) [27]
Surgical	Reoperation (e.g., foraminotomy, decompression)	Considered in persistent or worsening cases; outcomes vary	Lau & Park (2011) [28] Gu et al. (2014) [14]

Rehabilitation approaches

Rehabilitation plays a vital role in the management of C5 nerve palsy, regardless of the initial treatment strategy. Physical therapy is central, with emphasis on regaining strength in affected muscles through progressive resistance exercises, maintaining shoulder mobility, and preventing disuse atrophy [27,28]. Occupational therapy supports the development of compensatory strategies to enhance independence in daily activities. Electrical stimulation and neuromuscular facilitation techniques may be introduced in patients with severe weakness or delayed motor recovery, especially in the early stages. These interventions aim to activate motor units and maintain muscle tone while natural regeneration progresses (Table 4).

**Table 4:** Rehabilitation modalities for C5 palsy NMES: neuromuscular electrical stimulation; ROM: range of motion

Rehabilitation Component	Purpose	Application	Reference
Physical Therapy	Muscle strengthening, joint mobility	Active/resistive exercises for deltoid, biceps, shoulder stabilization	Kubota et al. (2021) [27]
Occupational Therapy	Promote independence in daily living	Training in adaptive tools, motor re-learning strategies	Kubota et al. (2021) [27]
Electrical Stimulation/NMES	Muscle activation in severe weakness	Stimulates contraction in paralyzed/weak muscles	Kubota et al. (2021) [27]
Early Mobilization	Prevent stiffness, improve circulation	Gentle ROM exercises; initiation within days post-surgery or symptom onset	Clinical consensus

Recovery timelines and long-term outcomes

The recovery trajectory for C5 palsy is highly variable (Table 5). Most patients show significant motor improvement within 6 to 12 months, though full recovery may take up to two years in some cases [23,29]. In a prospective study, approximately 54% of patients achieved complete motor recovery, with partial recovery in most remaining patients. Long-term functional outcomes are generally favorable, especially when rehabilitation is initiated early and sustained. However, a minority of patients may continue to experience residual weakness, particularly if rehabilitation is delayed or structural lesions persist. Follow-up care focusing on functional capacity, pain management, and psychological support is critical for optimizing long-term results [14,30].

**Table 5:** Recovery timelines and prognostic indicators

Timeframe	Expected Outcomes	Prognostic Factors	Key References
0-6 months	Initial motor improvements; recovery begins	Early rehab, absence of severe compression	Thompson et al. (2017) [23]
6-12 months	Significant recovery in strength and function	Structured physiotherapy adherence	Evaniew et al. (2022) [29]
12-24 months	Continued improvement; possible full recovery	Younger age, mild initial deficit	Saoud et al. (2013) [26], Gu et al. (2014) [14]
Long-term (2+ yrs)	Persistent deficits possible in some cases	Delayed rehab, severe initial palsy, comorbid stenosis	Seth et al. (2022) [30]

Discussion

The development of C5 nerve palsy following cervical spine surgery remains a significant postoperative complication, with varying incidence rates and contributing factors identified across studies. One of the critical factors associated with the development of C5 palsy is iatrogenic foraminal stenosis, particularly when lordotic correction is performed during posterior laminectomy and fusion procedures. Blizzard [31] highlighted that the correction of cervical lordosis, while intended to improve spinal alignment, can inadvertently lead to foraminal narrowing, thereby increasing the risk of nerve root compression and C5 palsy. Their study revealed that a narrower C4-C5 intervertebral foramen, caused by over-correction, correlates with a significantly higher risk of developing C5 palsy. In fact, iatrogenic foraminal stenosis was shown to elevate the incidence of C5 palsy by up to 20%, further emphasizing the importance of achieving a balanced alignment during surgical correction. This aligns with the findings of Pennington [32], who found that preoperative radiographic variables such as cervical curvature and foraminal stenosis could be used to predict postoperative C5 palsy with a reported predictive accuracy of 78%, suggesting that careful preoperative evaluation may reduce the risk.

In addition to surgical technique, the anatomical characteristics of patients play a substantial role in the susceptibility to C5 palsy. Lubelski et al [33] demonstrated that preoperative anatomical measurements, including the width of the C4-C5 foramen and the angle of the cervical curvature, were predictive of C5 nerve palsy. Their multivariate analysis of 150 patients undergoing posterior cervical decompression indicated that a narrower foramen was associated with an increased risk of nerve injury, and the risk was further exacerbated by the use of wide decompression techniques. Similarly, Nori et al [34] emphasized the importance of controlling the width of the laminectomy during surgery, suggesting that limiting the decompression area reduces the incidence of postoperative C5 palsy. Their findings, drawn from 263 patients undergoing muscle-preserving posterior decompression, found that a more limited decompression (particularly a width of less than 8 mm) resulted in a significantly lower rate of C5 palsy, supporting the need for precise and conservative surgical approaches.

Interestingly, some studies have focused on the role of hinge-sidedness in cervical laminoplasty. Levi et al. [21] investigated whether the hinge location in expansile open-door laminoplasty influences the laterality of C5 palsy. Their analysis showed no significant association between hinge-sidedness and the development of C5 palsy, suggesting that the traditional focus on hinge placement may not be as critical as other mechanical or anatomical factors. In contrast, Takemitsu [35] found that the incidence of C5 palsy was higher following laminoplasty procedures compared to posterior fusion, with C5 palsy rates approaching 15% in their cohort of 120 patients. This further supports the conclusion that the surgical technique itself, particularly laminoplasty, has a higher propensity for causing this complication, likely due to the greater degree of spinal cord manipulation and potential traction on the nerve roots during the procedure.

## Conclusions

C5 palsy is a complex and multifactorial complication that can occur after cervical spine surgery, influenced by a combination of surgical technique, patient anatomy, and preexisting spinal conditions. Key contributors include foraminal stenosis, cervical curvature abnormalities, and excessive spinal cord shift following decompression. The wide variation in incidence across procedures highlights the need for tailored surgical planning. Surgeons must carefully evaluate anatomical risk factors before selecting an approach. Individualizing treatment based on patient-specific characteristics can significantly reduce the likelihood of this complication. Preoperative imaging and risk assessment should play a central role in surgical decision-making. Minimizing nerve root traction and ensuring adequate decompression without overcorrection are critical preventive strategies. Future research should focus on integrating clinical, anatomical, and procedural data to better predict and prevent C5 palsy. Standardizing protocols across institutions may also help improve consistency in outcomes. Ultimately, a more personalized and precise approach to cervical spine surgery is key to reducing complications and improving patient recovery.
